# Back to School: Italian Teachers’ Perceptions of the Impact of COVID-19 on Personal and Social Well-Being and Teaching Methods

**DOI:** 10.3390/ijerph191811652

**Published:** 2022-09-15

**Authors:** Annamaria Porru, Raffaele Dicataldo, Irene Leo, Maja Roch, Daniela Lucangeli

**Affiliations:** Department of Developmental Psychology and Socialisation, University of Padova, 35131 Padova, Italy

**Keywords:** COVID-19, risk perception, job satisfaction, teaching methods, social relationships

## Abstract

During the COVID-19 pandemic, continuous closing and reopening of schools may have had an impact on teachers’ perception of the risk of contracting SARS-CoV-2 and of the effectiveness of health measures introduced to limit the spread of the virus, with consequences on teaching methods and relational bonds within schools. By means of an online survey, we measured: teachers’ stress, job-satisfaction, self-efficacy and emotions at work, risk-perception of contracting SARS-CoV-2, perception of effectiveness of health measures, teaching methods and social relationships. Participants were 2446 teachers (2142 women and 304 men) all engaged in the four educational stages. Most of the respondents were aged 50 or older (45%), followed by a group aged 41–50 (31%) and by a group aged <40 (24%). We used path analysis to test the impact that COVID-19 had, according to teachers, on teaching methods (Model 1) and social relationships (Model 2). In both models, teachers’ stress was positively directly associated with risk-perception of contracting SARS-CoV-2 (Model 1: *β* = 0.10; *p* < 0.001; Model 2: *β* = 0.09; *p* < 0.001). Additionally, we found an indirect path between teachers’ stress and risk-perception of contracting SARS-CoV-2 on the one hand, and perception of effectiveness of health measures on the other hand (Model 1: *β* = 0.02; *p* < 0.001; Model 2: *β* = 0.02; *p* < 0.001). These results suggest that, in emergencies, risk perception level, emotional regulation, and teachers’ stress levels were all key factors affecting teaching methods and relationship quality in schools.

## 1. Introduction

On 11 March 2020, the World Health Organization (WHO) declared a worldwide pandemic state of emergency (Public Health Emergency of International Concern—PHEIC). As regards the Italian context, the Decree of President of Council of Ministers (“D.P.C.M.”) dated 23 February 2020, introduced the first containment measures to be implemented in Italian schools; a.m. decree suspended the functioning of educational services in schools of all levels and degrees, including universities. Once schools opened again on 31 August 2020, the “Cts” (N. d. T.: “Italian scientific-technical Committee”) announced new containment measures, such as the use of personal protective equipment to be differently worn according to age. Containment measures required everyone to wear a mask and recommended some ordinary precautionary measures such as hand sanitization and a social distancing of at least one meter, while in more lively indoor places, a social distancing of at least two meters was recommended.

So far, many studies have focused on pandemic effects during lockdown, such as the impact on student achievement [1,2], on parenting [3,4] and on parental stress [5,6]. Many other studies have investigated pandemic effects on teaching methods [7], on teachers’ well-being during the COVID-19 pandemic [8,9], but to date still few studies have explored back-to-school-related effects on teachers’ well-being and their impact perception of the COVID-19 pandemic on social relationships and teaching methods.

Given the overwhelming effects of such a difficult time, experienced as a new dangerous situation in Italian schools, an investigation into this issue appeared to be necessary. The health emergency imposed for the first time in Italy, many changes in terms of teaching management, which probably affected, in their turn, several aspects of the school environment. Even once back at school, the new teaching methods used during lockdown have been partially maintained. Italian teachers had to face a new radical change employing the use of new teaching strategies as well as new technological tools.

The pandemic has set off a revolution in teaching practices and the allocation of resources for study and exercises supported not only by tools such as textbook websites, but also by Youtube and Raiplay channels [10].

Such activities involved a transposition of traditional teaching in telematics, whose use, being on average of 2–4 h per week [11], resulted in a high demand on teachers’ work. Accordingly, Italian teachers, like any other worker, had to face pressing and exhausting job requests.

Actually, recent research has shown that an increased use of digital technologies leads to a rise in work stress for any worker. The same research has also found a significant negative association between technostress and psychosocial demands, job satisfaction and self-efficacy with repercussions for daily work and quality of life [12].

Teachers’ quality of life plays a decisive role in making an education system stronger and to ensuring a population’s well-being. Teachers’ health and well-being seem to be key factors for supporting students’ well-being [13,14].

A recent study conducted in Italy has confirmed, in comparison to the rest of the population, teachers had a higher emotional burden to bear [15]. However, it seems necessary and essential to understand how teachers at different school levels have perceived these changes in terms of relations and teaching, the kind of changes that, unfortunately, can permanently affect pupils and teachers.

### 1.1. Variables Affecting Teachers’ Well-Being

Well-being at work relates to a subjective motivational sphere, self-efficacy and self-accomplishment included. Regarding the latter, even before the outbreak of COVID-19 pandemic, the subjective well-being of Italian teachers was deemed exposed to a significant risk of developing stress, as well as psychosomatic and psychological disorders [16,17].

With the aim to describe teachers’ well-being, Renshaw [18] singled out three key predictor variables namely: relationship quality within school, job-satisfaction, and self-efficacy. Another study has shown that: relational factors, work-related factors and above all, self-efficacy have an influence on teachers’ job satisfaction. These factors are deemed a guarantee of the teaching process quality as well as of collegial collaboration [19].

#### 1.1.1. Job-Satisfaction

These three indicators are so closely related to each other that teachers’ job-satisfaction could be considered as the result of emotional responses to their job and role [20,21]. As a matter of fact, job satisfaction, being directly influenced by the working atmosphere and interaction with colleagues and students, cannot be assessed as an independent factor [22,23,24].

Skaalvik and Skaalvik [23] observed the relationship between teachers’ perception and their feeling of belonging, emotional exhaustion, job satisfaction and motivation to leave the teaching profession. The findings showed that value-consonance, as well as supervisory support and positive relations with colleagues and parents, were predictive of a feeling of belonging, while time pressure and discipline problems were predictive of emotional exhaustion. Moreover, both a feeling of belonging and emotional exhaustion were closely related to job satisfaction, while the negative association between emotional exhaustion and job satisfaction was predictive of motivation to leave the teaching profession.

#### 1.1.2. Self-Efficacy

Caprara [25] believed job satisfaction to be a “main determinant” of attitudes and performance and considered self-efficacy a key promoter of teachers’ job satisfaction. Teachers report that job satisfaction fosters daily classroom activities, such as work with students, observation of their progress and cooperation with supportive colleagues, the whole to the advantage of the general working climate. High self-efficacy levels lead to an enhanced commitment and involvement in day-to-day activities. Teachers unsatisfied with their job are less committed and are more motivated to leave the teaching profession [26].

In primary school [27] as well as in lower and upper secondary school [25,28,29], it was proven that teachers’ self-efficacy makes them more satisfied with their job and relationships, compared to less efficient colleagues.

Several studies pointed out the relationship existing between job satisfaction and self-efficacy [21,30,31]; they practically demonstrated how teachers, having low self-efficacy levels, experience greater teaching difficulties with a following rise in stress levels [32] and a lowering of job satisfaction levels [33,34].

Studies [35,36] conducted on teachers proved the existence of a negative correlation between high self-efficacy levels and low emotional exhaustion and depersonalization levels. Teachers having high self-efficacy levels when implementing teaching strategies and managing classrooms are generally less likely to feel emotionally exhausted and to depersonalize their students, compared to teachers with low self-efficacy levels [37,38].

The same correlation was found among senior teachers: those with a higher sense of self-efficacy experienced lower feelings of burnout [39,40]. Some studies’ results suggest that teachers working with higher self-efficacy experience less work-related stress [32,41]. Even when teachers are confident in their own skills and use them to engage students actively and to manage students’ misconduct, they feel more self-accomplished and less emotionally exhausted.

#### 1.1.3. Emotions

This is one of the reasons why, even in the teaching context, emotions are mainly perceived as part of a process, through which teachers try to inhibit, encourage and manage the display of their feelings and emotions, according to professional norms expectations and beliefs [42]. Emotional management requires implementation of specific strategies.

Teaching means triggering emotional responses [43], accordingly, emotions are not only a source of useful information for the agency, but also an investment form for teachers in terms of identity and values.

Teachers invest energy and emotions in their job, often blending their sense of personal and professional identity, in a way that makes schools and classrooms places where they can fulfil themselves [44]. Teachers’ identity, well-being and efficacy provide the emotional setting for teaching.

Several studies emphasized how teachers’ emotional intelligence has a powerful impact on job-satisfaction [45,46,47,48].

According to Jennett, Harris and Mesibov [49] all teachers experience work-related stress, a kind of stress which mainly depends on how teachers respond and adapt themselves to various tasks they have to perform every day, and to actions they suffer, having potential harmful effects on physiological, psychological and behavioral levels. Most of the teachers successfully deal with work-related stress in many ways, such as for example through proactive problem-solving, colleagues’ social and emotional support, reorganization of teaching conditions, cooperation with parents or a change in their teaching methods. Nevertheless, inadequate stress management can make stress become chronic and result in burnout [49,50,51].

### 1.2. Risk Perception

Risk perception is defined as a subjective psychological assessment connected to the understanding of risk events, probability and consequences [52,53]. It is proven that people who have experienced natural disasters have a high perception of environmental risk and are more likely to easily remember the catastrophic [52,54,55,56]. We have also found that lockdown, quarantine, and isolation resulting from uninterrupted pandemic escalation have led people to become worried, anxious, and constantly perceiving the risk of contracting COVID-19 [57]. Several studies on risk perception have recommended the inclusion of emotional and social variables that directly or indirectly influence subjective perception [52,58,59].

#### 1.2.1. Teachers’ Risk Perception during Pandemic

It remains unexplored how teachers experienced the perception of the risk of contracting COVID-19.

In September 2020, in Italy, some of the factors so far described were once again modified because of the controversial social and political debate due to schools reopening.

The spread of COVID-19 forced teachers to adjust their teaching methods and, at the same time, face unexpected challenges. As far as the Italian context is concerned, the most extensive and precise research into the pandemic’s impact on school settings was provided in 2020 by SIRD (N. d. T.: “Società Italiana di Ricerca Didattica”, “Italian Educational Research Society”) [10,60,61]. The research highlighted weaknesses related to distance teaching, management of school facilities, students’ engagement, as well as strengths resulting from cooperation with school leadership and families, especially in pre-primary and primary schools [62].

The perceived risk of contracting SARS-CoV-2 is associated with a range of negative emotions (such as anxiety and depression) that are configured as mediating factors of psychological well-being [63]. Sociodemographic factors, such as gender or the place where the school is located, also seem to have an influence on the emergence of anxiety symptoms [63].

Risk perception consists of two elements, namely: personal vulnerability perceived vis-à-vis the situation and probability that a threat will occur [64,65]. Teachers having a higher perception of risk were more willing to comply with protection measures. However, women teachers above all, adopted preventive measures and experienced less anxiety [66,67,68]. During the COVID-19 pandemic, anxiety symptoms appeared to be prevailing among women teachers, while they were less frequent among teachers working in schools located far from major urban centers [69]. However, studies have shown that teachers with a higher perception of risk showed less self-efficacy [70]. Based on the literature we examined, there seems to be the lack of a theoretical construct that can facilitate the understanding of how the perception of risk directly or indirectly influences teaching.

School managers were responsible for any decisions aimed at avoiding classroom gatherings in many ways: by splitting up classes into several groups with different time slots for lessons, by adjusting the whole timetable as well as the total amount of hours spent for individual discipline. Several studies showed that fear prompted teachers to accept COVID-19 protective measures, despite their perception of deficient resources [71,72].

Even before the outbreak of the COVID-19 pandemic, the teaching profession was described as a job involving many stressors and difficulties [73]. Teachers cope with various “requests” ranging from managing a classroom [74] to political changes, influencing teaching methods and students’ assessment [75], to workload [76,77]. During the COVID-19 pandemic, stress caused by sudden change from conventional to on-line teaching methods, without even providing teachers with the necessary training, helped to make difficulties worse [78]. It should be considered that teachers had to face not only an unexpected change in teaching methods, strains due to the COVID-19 pandemic itself, digital teaching from home, management of students’ relationships online, but also health-related problems.

#### 1.2.2. The Effects of COVID-19 on Different School Levels

Several studies have explored the effect the pandemic has had on different school levels.

The pandemic’s impact has clearly affected every school level and it has been felt in all schools and by all teachers.

German and Chinese secondary school teachers experienced higher stress levels, when compared to special educational class teachers, who suffered lower stress levels [69,79]; in Spain pre-primary and primary school teachers are those who showed the highest stress levels [80]. Specifically, women teachers were more stressed than men teachers, while at the same time women teachers more often took advantage of effective coping strategies, compared to their male colleagues [69,79].

Bondioli and Savio, ref. [62] conducted a survey revealing that nursery and pre-primary teachers, under health constraints, experienced communication difficulties, which had an impact on a social-relational level.

A survey conducted in Canada [81] demonstrated that teachers’ positive attitudes toward change (cognitive, emotional, and behavioral ones), positive perception of administrative support, teaching efficacy and positive attitudes toward the use of technology were successfully correlated with each other and with teachers’ resilience. In addition, research conducted on an Italian sample [7], showed that teachers apparently perceived significant emotional distress related to negative and worrying events which led teachers to emotional and psychological exhaustion with the beginning of new school year in September 2020. Ozamiz-Etxebarria [80] confirmed that a high percentage of Spanish teachers showed symptoms such as anxiety, stress, and depression at the time when schools and universities were reopened.

### 1.3. Aims and Hypotheses

The lack of a basic construct to understand teachers’ responses to an emergency, led us to analyze relationships with numerous variables that could be related and which were presented in the introduction. We tried to verify if emergency and teachers’ personal factors could have an effect on the change of teaching methods and relationships.

This study aimed to answer the following research questions:

(1) We intended to verify the existence of a positive and significant correlation between our independent variables and dependent variables. Specifically, we asked ourselves:
  (a) Is there any significant and positive correlation between social relationships at school (ISR) and teachers’ stress (TS), job satisfaction (JS), self-efficacy (SE), and emotional intensity at work (EMO)?
  (b) Is there any significant and positive correlation between teaching methods (ITM) and teachers’ stress (TS), job satisfaction (JS), self-efficacy (SE), and emotional intensity at work (EMO)?

We analyzed four dimensions: teachers’ stress (TS), job satisfaction (JS), self-efficacy (SE) and emotional intensity at work (EMO), with the aim of investigate whether these stress and cognitive factors (self-efficacy, emotions, and job satisfaction) have had any effect on dependent variables: teaching methods (ITM) and social relationships at school (ISR);

(2) We intended to verify the existence of a causal link between our outcome and the constructs examined. In fact, we expected that during the pandemic the link between the outcome and constructs had been mediated by teachers’ perception of risk of contracting SARS-CoV-2 (CRP) and perception of the effectiveness of health measures (PEHM). We have therefore answered the following questions:
  (a) Is there any causal link between social relationships at school (ISR) and the constructs of teachers’ stress (TS), job satisfaction (JS), self-efficacy (SE), and emotional intensity at work (EMO)? During the pandemic this link was mediated by variables such as: teachers’ perception of risk of contracting SARS-CoV-2 (CRP) and effectiveness perception of health measures (PEHM)?
  (b) Is there any causal link between teaching methods (ITM) and the constructs of teachers’ stress (TS), job satisfaction (JS), self-efficacy (SE), and emotional intensity at work (EMO)? During the pandemic period this link was mediated by variables such as: teachers’ risk perception of contracting SARS-CoV-2 (CRP) and perception of the effectiveness of health measures (PEHM)?

Although the literature clarifies the relationship existing between stressors and personal cognitive factors, so far no one has tried to explain the global relationship between all these variables. Our research tries to examine the predictive relationship between the intervening variables (teachers’ stress (TS), job satisfaction (JS), self-efficacy (SE) and emotions intensity at work (EMO) through two models of path analysis on teaching methods (ITM) and social relationships (ISR);

(3) Finally, we examined and tested whether both models differed significantly in terms of structural parameters across school levels and teachers’ age. We more likely expected to find a different sensitivity in the primary school group than in the secondary school group and that an older age could have greater negative effects on teaching and relationships. We have previously mentioned that several investigations verified the COVID-19 pandemic effects on different school levels, but so far, no one has proved whether those effects differed according to school levels and teachers’ ages. Our research tries to explain the two relationships existing between variables in subgroups created according to different school levels and to teachers’ ages.

## 2. Materials and Methods

### 2.1. Study Design

We conducted a cross-sectional quantitative study, with non-random convenience sampling of teachers. By means of an online survey, we assessed teachers’ perception of risk of contracting SARS-CoV-2, their perception of the efficacy of the health measures introduced in schools, and their opinions on COVID-19 impacts on social relationships and teaching methods within the school. Additionally, we assessed teachers’ stress, self-efficacy, job satisfaction, and intensity of emotions experienced during classroom practice. The sample was recruited by sending an email to the schools and teachers enrolled in the municipal lists. The purposes of the research reported in the informed consent were explained in the email. We have chosen convenience sampling for the sampling technique so that the participants are selected because they are often readily and easily available. It is an inexpensive and easy option compared to other sampling techniques [82]. Data collection took place between December 2020 and February 2021, and throughout that period, the online survey was permanently accessible.

### 2.2. Participants

A total of 3518 teachers attempted to complete the survey, but only 2446 (2142 women and 304 men) finished. For the following analyses, only those questionnaires completed in all the sections provided were considered valid and retained. As regards the sample size, [83] it suggested that a minimum of 10–20 subjects per parameter estimated in the model was optimal. However, the sample size of 2446 respondents exceeded that number, thus making the analysis justified. Of the 2446 respondents (see Table 1), about 88% of the teachers were female and 12% male. Most of the respondents were aged 50 or older (45%), followed by a 41–50 years old group (31%) and then by the <40 years old group (24%). Moreover, most of the respondents had a master’s degree (56%), followed by those having a high school degree (24%), specialization after a master’s degree (14%), and a bachelor’s degree (6%). As regards teachers’ experience (Mage = 18.2 years; SD = 11), most of them had over 21 years of experience (40%), 30% had between 11 and 20 years, 15% between 5 and 10 years and 15% had under 5 years. Among the respondents, about 13% worked in preschool, 34% in primary school, 24% in lower secondary and 29% in higher secondary. Most of them were curricular teachers (86%), while the remaining 14% were special needs teachers. Another aspect we analyzed referred to the geographic area of teaching. We noticed that the majority of those who completed the questionnaire worked in northern Italy (64%), followed by southern (19%) and central Italy (17%). The last aspect we analyzed concerned the implementation of the distance education (DE). Most respondents (59%) stated that at the time of survey completion, DE was implemented in their schools. In addition, the percentage of DE hours was very variable: in about 60% of cases, education was carried out completely at distance, 10% from 25% to 50%, 10% from 50% to 75% and for the remaining 20% the proportion of DE hours was less than 25% of the total.

### 2.3. Survey Description and Outcomes

The survey was designed to capture, as well as possible, the following eight constructs. The primary outcomes assessed through the brand-new items were teachers’ perceptions about the impact of COVID-19 on teaching methods and school relationships (ITM; ISR). Other constructs, namely teachers’ stress (TS), job satisfaction (JS), self-efficacy (SE) and emotional intensity at work (EMO) were measured through standardized teachers’ questionnaires. Additionally, through several items, we measured teachers’ perception of risk of contracting SARS-CoV-2 (CRP) and perception of the effectiveness of health measures (PEHM).

The survey consisted of 122 items divided in two sections. The first section captured demographic information of the respondents, i.e., gender, age, experience, teaching level, and teaching location.

The second sections consisted of 117 items capturing the following 8 constructs, in detail:

The K10 [84] was used to assess teachers’ psychological distress. The K10 had ten questions regarding symptoms experienced during the last 30 days; each item had five possible responses from “all the time” to “never” scored from five to one, so the total score could vary from a minimum of ten to a maximum of fifty (α = 0.89).

The Metacognitive Questionnaires for Teachers [84] were used. It is a battery of self-reporting questionnaires, which include job satisfaction, teaching practices, emotions in teaching, which includes positive and negative emotions during teaching and positive and negative emotions as a teacher, teaching strategies, self-efficacy in teaching and incremental beliefs. For our research aims, we used only a few subscales: job satisfaction, five 7-point items (α = 0.80); emotional intensity in teaching, thirty 5-point items (α = 0.79); and self-efficacy in teaching, twenty-four 9-point items (α = 0.96).

In addition, we developed 12 items to measure teachers’ perceptions of their risk of contracting SARS-CoV-2 (CRP) and their perceptions about the effectiveness of health measures (PEHM). For CRP we asked teachers to rate the likelihood of contracting COVID-19 with respect to age, gender, and hours worked. Each item has ten possible responses from “not at all likely” to “extremely likely” scored from 1 to 10, so the total score has a minimum of 12 and maximum of 120 (α = 0.88). For PEHM we asked teachers to evaluate the effectiveness of some sanitary measures introduced at school to limit the spread of the virus (wearing masks; hand sanitization etc.). Each item has ten possible responses from “not at all” to “extremely” scored from 1 to 10, so the total score has a minimum of 6 and maximum of 60 (α = 0.87).

At the end of this section, there were 36 questions allowing teachers to rate the pandemic’s effect on social relationships (ISR) and teaching methods (ITM). For ISR we asked teachers to rate the impact that the pandemic has had on their relationships with students, colleagues and families and on the relationships among students. Each item had ten possible responses from “anyhow” to “a lot” scored from 1 to 10, so the total score varied from a minimum of 4 to a maximum of 40 (α = 0.87). For ITM we asked teachers to rate the impact that the pandemic has had on their teaching methods. Each item had ten possible responses from “anyhow” to “a lot” scored from 1 to 10, so the total score varied from a minimum of 14 to a maximum of 140 (α = 0.95).

### 2.4. Procedure

Participants were recruited through email announcements. School boards were directly contacted, and the link sent to participants via email distribution lists. A snowballing system was encouraged, asking participants to share the survey with other teachers. Prior to participating in the survey, participants received a description and the objectives of the study. A total of 2446 individuals attempted to complete the survey. Written informed consent was obtained from all the participants prior to study enrolment. This study was approved by the Ethics Committee of the University of Padua (protocol number: 3887) and performed in accordance with the principles expressed in the 1964 Declaration of Helsinki.

The data collection took place in accordance with the European Data Protection Regulations. It was performed by means of a structured, anonymous, self-administered, online survey using the Qualtrics Experience Management, a secure web application for building and managing online surveys and databases. The data were downloaded from Qualtrics into MS Excel and verified for coding accuracy. Given that in Qualtrics we had the option to make the answers mandatory, the database was complete, and no data were missing.

### 2.5. Statistical Analyses and Models Description

Descriptive statistical analyses were performed in IBM SPSS Statistics. A total score for each construct was calculated by summing the scores obtained within items in each subscale. Each construct was thus summarized by a single observed measure therefore, path analysis using structural equation modelling (SEM) was the primary data analytic strategy. R package lavaan, version 0.4-11 (R Fondation for Statistical Computing, c/o Institute for Statistics and Mathematics, Vienna, Austria) [85], was used to evaluate and analyze our models (described below).

The main aim was to examine relationships of teachers’ stress (TS), job satisfaction (JS), self-efficacy (SE), teachers’ emotional intensity (EMO), teachers’ perception of risk of contracting SARS-CoV-2 (CRP) and perception of the effectiveness of health measures (PEHM) on two separate dependent variables using path analysis and the model fit indexes. The estimation of the model consists of obtaining some idea of the parameters that compose the reproduced matrix, so that they are similar to those in the initial matrix. The identification and interpretation of the fit indexes resulting from the estimation of the model allow us to draw some conclusions regarding the tested model. Model fits were evaluated by using the following multiple indices: chi-square statistics, comparative fit index (CFI), Akaike’s information criterion (AIC), Bayesian information criterion (BIC), root mean square error of approximation (RMSEA), and standardized root mean square residuals (SRMR). Typically, RMSEA values below 0.08, and CFI values equal to or greater than 0.95, and SRMR equal to or less than 0.10 indicate an acceptable model fit [86].

Since no specific theoretical models exist concerning how these variables are related to the impact that COVID-19 had on teaching methods (ITM) and school social relationships (ISR) during the pandemic, based on a literature review, we tested models in which all direct and indirect relations were allowed from predictors and dependent variables.

**Figure 1:**
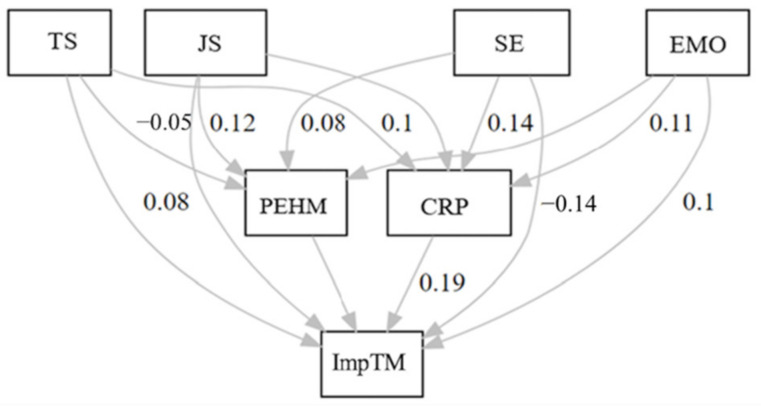
Model 1: COVID-19 pandemic impacts on teaching methods. Note: TS = teachers’ stress, JS = job satisfaction, SE = Self-efficacy, EMO = emotional intensity at work, CRP = perception of risk of contracting SARS-CoV-2; PEHM = Perception of the effectiveness of health measures; ImpTM: impact of COVID-19 on teaching methods. Only coefficients of statistically significant paths (solid lines) are reported.

**Figure 2:**
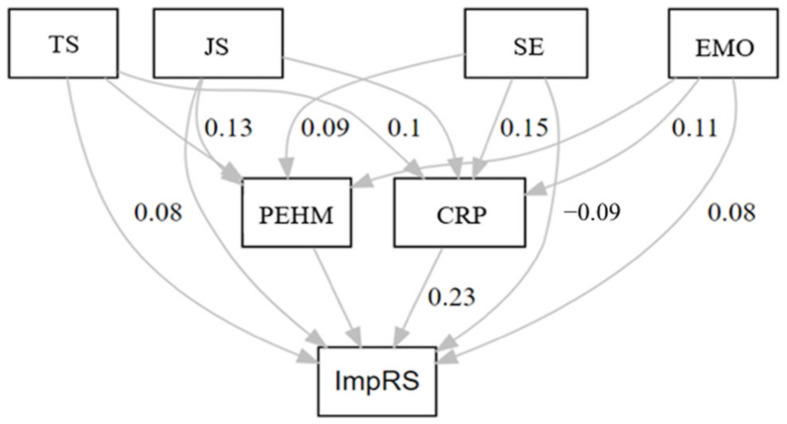
Model 2: COVID-19 pandemic impacts on social relationships. Note: TS = teachers’ stress, JS = job satisfaction, SE = Self-efficacy, EMO = emotional intensity at work, CRP = perception of risk of contracting SARS-CoV-2; PEHM = Perception of the effectiveness of health measures; ImpRS: impact of COVID-19 on relationships at school. Only coefficients of statistically significant paths (solid lines) are reported.

The arrows that link predictors such as TS, JS, SE and EMO and CRP, PEHM to teachers’ perception about the impact of the COVID-19 pandemic on teaching methods (Model 1) and relations (Model 2) represent causal relationships in the direction of the arrows (see Figure 1 and Figure 2). The analytical process was started by testing the hypothesized models. Thus, beginning with total models, we tested these in different subgroups. Additionally, we tested the models’ invariance across school levels and teachers’ age. To facilitate the reading of the results, after the first descriptive analysis, we organized two separate paragraphs, one for each dependent variable.

## 3. Results

### 3.1. Descriptive Statistics

Table 2 shows descriptive statistics of variables inserted in the following models and correlations among variables for the whole sample.

On average, teachers report that the pandemic could substantially affect teaching methods and social relationships. Teachers report high levels of self-efficacy in teaching and an average score on emotional intensity experienced while teaching. Low scores on job satisfaction, show that teachers, on average, are dissatisfied with the work they do, particularly during the pandemic, however their low scores on Stress indicate that they were not experiencing significant feelings of distress at the time the survey was completed. Despite reporting quite a high score on the perceived risk of contracting SARS-CoV-2, teachers perceive the health measures introduced to limit the spread of the virus as fairly effective.

Correlations among the two main outcomes and predictors show that the more teachers are stressed, the more they rate the impact on teaching methods and on relationships (*r* = 0.17; 0.16); the more teachers are satisfied at work, the less they rate the impact on teaching methods and on relationships (*r* = −0.11; −0.10); the more effective teachers feel in their work, the less they rate the impact on teaching methods and on relationships (*r* = −0.12; −0.08); the more intensely teachers feel emotions and perceive the risk of contracting COVID 19, the greater the impact they believe the pandemic has had on teaching and social relationships at school (respectively *r* = 0.14; 0.13; *r* = 0.20; 0.24).

### 3.2. Path Analysis

We tested two separate models, with the same predictors and a different dependent variable. Models were tested on the total sample end in different subgroups. Additionally, we tested model invariance, using multi-group path analysis, across school levels, teachers’ ages and teaching locations, to examine whether differences in parameters and relations between variables across groups, were statistically significant.

#### 3.2.1. Path Analysis with Teachers’ Perception about the Impact of COVID-19 on Teaching Methods as Dependent Variable

In Model 1, we analyze the causal relationships between teachers’ stress (TS), job satisfaction (JS), self-efficacy (SE), emotional intensity (EMO), risk perception of contracting SARS-CoV-2 (CRP) and perception of the effectiveness of health measures (PEHM) to teachers’ perception about the impact of COVID-19 on teaching, upon being back at school. Table 3 shows fit indexes of Model 1 for the whole sample (*N* = 2015) and for each subgroup, namely school level and teacher age.

Path analysis results indicated that Model 1, for the whole sample, had good fit to the data (*χ*^2^ = 1958, df = 3, *p* < 0.058, CFI = 1, NNFI = 1, RMSEA = 0.0000). Completely standardized path coefficients of Model 1, for the whole sample, are reported in Figure 1.

Teachers’ stress (TS) was positively, directly associated with teachers’ perception of risk of contracting SARS-CoV-2 (*β* = 0.10; *p* < 0.001), negatively directly associated with the perception of the effectiveness of health measures (*β* = −0.05; *p* < 0.05) and positively directly associated with teachers’ perceptions about the impact of COVID-19 on teaching (*β* = 0.07; *p* < 0.01). Additionally, we found an indirect path between TS and teachers’ perceptions about the impact of COVID-19 through teachers’ perception of risk of contracting SARS-CoV-2 (CRP) and perception of the effectiveness of health measures (PEHM) (*β* = 0.02; *p* < 0.001). Teachers’ job satisfaction (JS) was positively, directly associated with perception of the effectiveness of health measures (*β* = 0.12; *p* < 0.001). Teachers’ self-efficacy (SE) was positively directly associated with teachers’ perception of risk of contracting SARS-CoV-2 (*β* = 0.13; *p* < 0.001), and perception of the effectiveness of health measures (*β* = 0.08; *p* < 0.01) and negatively directly associated with teachers’ perceptions about the impact of COVID-19 on teaching (*β* = −0.14; *p* < 0.001). Additionally, we found an indirect path between SE and teachers’ perception about the impact of COVID-19 through teachers’ perception of risk of contracting SARS-CoV-2 (CRP) and perception of the effectiveness of health measures (PEHM) (*β* = 0.03; *p* < 0.001). Teachers’ emotional intensity at work (EMO) was positively directly associated with their perception of the risk of contracting SARS-CoV-2 (*β* = 0.10; *p* < 0.001) and with teachers’ perception about the impact of COVID-19 on teaching (*β* = 0.19; *p* < 0.001). Additionally, we found an indirect path between EMO and teachers’ perception about the impact of COVID-19 through teachers’ perception of risk of contracting SARS-CoV-2 (CRP) and perception of the effectiveness of health measures (PEHM) (*β* = 0.02; *p* < 0.001). Eventually, we found that teachers’ perception of risk of contracting SARS-CoV-2 (CRP) was positively, directly associated with teachers’ perception about the impact of COVID-19 on teaching (*β* = 0.19; *p* < 0.001). Overall, Model 1 explains a percentage of variance of the dependent variable, namely teachers’ perception about the impact of COVID-19 on teaching, equal to 9%.

As reported in Table 3, Model 1 has good fit for the whole sample as well as for the subgroups analyzed, namely school levels and teachers’ ages.

#### 3.2.2. Multigroup Path Analysis

Multi-group path analysis was employed to examine and test whether in Model 1 there were significant differences in the structural parameters across school levels and teacher age. Three models were compared to test cross-group invariance: (a) a baseline model with no equality constraints (i.e., the model structure was equal across the groups); (b) a constrained model where all parameters were constrained to be equal between the groups, and (c) partial invariance where some parameters have been freed. The fits of these models were then compared using a chi-square difference test. If the chi-square test for difference is significant, then structural paths for groups are non-invariant. In Table 4, we reported results from multi-group path analysis of Model 1 in different subgroups.

As can be seen in Table 4, Baseline models provided adequate fit indexes to the data in each subgroup, showing that Model 1 reproduces the relationships between variables well in all subgroups. Once there configural invariance we tested metric invariance in which all parameters were constrained to be equal between the groups. The constrained model provided a sufficient fit for the data as well. However, constraining the parameters to be equal resulted in a decrease in model fit. If metric invariance is rejected, it means that the parameters are different across groups and thus it becomes important to determine which relationship(s) differ across groups analyzing the partial metric invariance. Below, we describe the parameters/relationships freed up and reported parameter estimates from the path analysis across subgroups.

In detail, concerning school levels, three relationships have been released: that between TS and CRP, between TS and PEHM and between EMO and teachers’ perception about the impact of COVID-19 on teaching. We found that teachers’ stress (TS) was positively and directly associated with teachers’ risk perception of contracting SARS-CoV-2 only for teachers working in primary schools and lower secondary schools (respectively *β* = 0.15 and *β* = 0.16; *p* < 0.001), whereas this relationship was not significant for teachers working in higher secondary schools. The significant and negative association between TS and PEHM remains statistically significant only for teachers working in Primary schools (*β* = −0.10; *p* < 0.01). Finally, we found that teachers’ emotions at work (EMO) was positively directly associated with their perception about the impact of COVID-19 on teaching only for teachers working in primary schools (*β* = 0.12; *p* < 0.001).

Regarding teachers’ age, we found that Model 1 was invariant in each subgroup and therefore the relationships between the variables are similar across the three age groups.

#### 3.2.3. Path Analysis with Teachers’ Perception about the Impact of COVID-19 on Social Relationships, upon Being Back in School, as Dependent Variables

In Model 2, we analyze the causal relationships between teachers’ stress (TS), job satisfaction (JS), self-efficacy (SE), emotional intensity at work (EMO), perception of risk of contracting SARS-CoV-2 (CRP) and perception of the effectiveness of health measures (PEHM) to teachers’ perception about the impact of COVID-19 on social relationships, upon being back in school. Table 5 shows fit indexes of Model 2 for the whole sample (*N* = 2351) and for each subgroup.

Path analysis results indicated that Model 2, for the whole sample, had a good fit to the data (*χ*^2^ = 1486, df = 3, *p* < 0.685, CFI = 1, NNFI = 1, RMSEA = 0.0000). Completely standardized path coefficients of Model 2, for the whole sample, are reported in Figure 2.

Teachers’ stress (TS) was positively, directly associated with teachers’ perception of risk of contracting SARS-CoV-2 (*β* = 0.09; *p* < 0.001), and with teachers’ perception about the impact of COVID-19 on social relationships (*β* = 0.08; *p* < 0.001). Additionally, we found an indirect path between TS and teachers’ perceptions about the impact of COVID-19 through teachers’ perception of risk of contracting SARS-CoV-2 (CRP) and perception of the effectiveness of health measures (PEHM) (*β* = 0.02; *p* < 0.001). Teachers’ job satisfaction (JS) was positively, directly associated with perception of the effectiveness of health measures (*β* = 0.13; *p* < 0.001). Teachers’ self-efficacy (SE) was positively, directly associated with teachers’ perception of risk of contracting SARS-CoV-2 (*β* = 0.15; *p* < 0.001), and perception of the effectiveness of health measures (*β* = 0.09; *p* < 0.001) and negatively, directly associated with teachers’ perceptions about the impact of COVID-19 on social relations (*β* = −0.09; *p* < 0.001). Additionally, we found an indirect path between SE and teachers’ perceptions about the impact of COVID-19 through teachers’ perception of risk of contracting SARS-CoV-2 (CRP) and perception of the effectiveness of health measures (PEHM) (*β* = 0.03; *p* < 0.001). Teachers’ emotional intensity at work (EMO) was positively directly associated with their perception of risk of contracting SARS-CoV-2 (*β* = 0.11; *p* < 0.001) and with teachers’ perceptions about the impact of COVID-19 on social relationships (*β* = 0.07; *p* < 0.001). Additionally, we found an indirect path between EMO and teachers’ perception about the impact of COVID-19 through teachers’ perception of risk of contracting SARS-CoV-2 (CRP) and perception of the effectiveness of health measures (PEHM) (*β* = 0.03; *p* < 0.001). Eventually, we found that teachers’ perception of risk of contracting SARS-CoV-2 (CRP) was positively, directly associated with teachers’ perception about the impact of COVID-19 on social relationships (*β* = 0.23; *p* < 0.001). Overall, Model 2 explains a percentage of variance of the dependent variable, namely teachers’ perception about the impact of COVID-19 on relations, equal to 9%.

As reported in Table 5, Model 2 has a good fit for the whole sample as well as for the subgroups analyzed, namely school levels, teacher age and teaching locations. However, Model 2, for high school teachers, has borderline fit indices.

#### 3.2.4. Multigroup Path Analysis

Multi-group path analysis was employed to examine and test whether in Model 2 there were significant differences in the structural parameters across school levels and teacher age. The same procedure used for the Model 1 has been used for the following analyses. In Table 6, we reported results from multi-group path analysis of Model 2 in different subgroups.

In detail, concerning School levels, six relationships have been released: that between TS and CRP, TS and PEHM, SE and CRP, EMO and PEHM and those between JS and SE with teachers’ perception about the impact of COVID-19 on social relationships.

We found that teachers’ stress (TS) was positively and directly associated with teachers’ perception of risk of contracting SARS-CoV-2 for teachers working in preschools (*β* = 0.14; *p* < 0.05), primary schools (*β* = 0.15; *p* < 0.001) and lower secondary schools (*β* = 0.16; *p* < 0.001) whereas this relationship was not significant for teachers working in higher secondary schools. The significant and negative association between TS and PEHM remains statistically significant only for teachers working in primary schools (*β* = −0.10; *p* < 0.01).

SE was positively and directly associated with teachers’ perception of risk of contracting SARS-CoV-2 at every school level, with a higher relation in preschools (*β* = 0.27; *p* < 0.001) that decreases in primary schools, lower and higher secondary schools (respectively *β* = 0.13; *p* < 0.001; *β* = 0.12; *p* < 0.01; *β* = 0.15; *p* < 0.001).

We found that teachers’ emotional intensity at work (EMO) was positively directly associated with PEHM only for teachers working in preschools (*β* = 0.11; *p* < 0.05). Eventually, SE was negatively and directly associated with teachers’ perception about the impact of COVID-19 on relations for teachers in primary schools, lower and higher secondary schools (respectively *β* = −0.07; *p* < 0.05; *β* = −0.16; *p* < 0.001; *β* = −0.09; *p* < 0.05) whether JS was negatively associated with teachers’ perception about the impact of COVID-19 on social relations only in preschools (*β* = −0.15; *p* < 0.001).

Regarding teacher age, three relationships have been realized: that between TS and CRP, and those between JS and CRP with teachers’ perception about the impact of COVID-19 pandemic on relations. We found that TS was positively and directly associated with teachers’ perception of risk of contracting SARS-CoV-2 for teachers between 40 and 50 years (*β* = 0.07; *p* < 0.05), and teachers older than 50 years (*β* = 0.14; *p* < 0.001), whereas this relationship was not significant for younger teachers. Additionally, SE was negatively and directly associated with teachers’ perceptions about the impact of COVID-19 on relations only for teachers between 40 and 50 years (*β* = −0.11; *p* < 0.01), and teachers older than 50 years (*β* = −0.09; *p* < 0.001). Eventually, we found that as the age of teachers increases, the strength of the relationship between CRP and teachers’ perceptions of the impact of COVID-19 on relationships increases (respectively *β* = 0.18; 0.20; 0.24; *p* < 0.001).

## 4. Discussion

The current study provides data regarding teachers’ perception on how different personal aspects, as well as contextual aspects related to the containment of the COVID-19 pandemic, influenced teaching methods and relationship quality in schools after the first wave of lockdowns in Italy.

To achieve our aim, we tested a theoretical model set out to examine the relationships existing between several factors, such as teachers’ stress (TS), job satisfaction (JS), self-efficacy (SE), teachers’ emotional intensity (EMO), teachers’ perception of risk of contracting SARS-CoV-2 (CRP) and perception of the effectiveness of health measures (PEHM) on two separate dependent variables: teaching methods (ITM) and social relationships at school (ISR). To do this we used path analysis and the model fit indexes. Initial hypotheses 1 and 2 were verified and fulfilled, while hypothesis 3 was not confirmed. In the following paragraphs, we will discuss the results of these 3 hypotheses.

As previously mentioned, various research has shown the effects that teachers’ stress (TS), job satisfaction (JS), self-efficacy (SE), teachers’ emotional intent (EMO) have on teaching and relationship quality in schools. The innovative aspect of our research is to have combined these variables with two theoretical models to explain how, during the pandemic period, these variables had a decisive impact on teaching methods and relationship quality.

### 4.1. COVID-19 Pandemic Impacts on Teaching Methods

Model 1 tries to verify the indirect path between TS, JS, SE, EMO and teachers’ perception of COVID-19’s impact through teachers’ risk perception of contracting SARS-CoV-2 (CRP) and perception of the effectiveness of health measures (PEHM) on teaching methods (ITM).

Our descriptive analysis confirms the existence of a positive association between TS and CRP.

Previous research carried out among Italian health professionals showed that teachers displayed higher levels of stress, which reflects the perceived risk of infection. Stress was related to an increase in risk perception [87].

Furthermore, a higher level of TS implies a lower perception of health measures effectiveness.

Similarly, increased EMO reveals a greater perception of the risk of contracting SARS-CoV-2 (CRP) among teachers and a greater impact on teaching quality in schools.

These results are consistent with research on an Italian sample showing that, in the case of high levels of perceived risk, Italians faced COVID-related news, the lockdown experience and restrictive measures in an “emotional” way combined with a feeling of fear, anger and sadness [88].

Differently, as far as our model, we have observed an indirect path between EMO and SE and teachers’ perception of the impact of COVID-19 through teachers’ risk perception of contracting SARS-CoV-2 (CRP).

This factor leads us to reflect on the importance of coping strategies that might improve one’s response to an emergency. Indeed, several reports have shown that coping strategies mediate between burnout and well-being in the workplace [89,90,91].

### 4.2. COVID-19 Pandemic Impacts on Social Relationships

Model 2 tries to verify the indirect path between TS, JS, SE, EMO and teachers’ perception of COVID-19’s impact on relations through teachers’ risk perception of contracting SARS-CoV-2 (CRP) and perception of health measures effectiveness (PEHM) on social relationships at school (ISR). Our analysis shows the existence of a positive association between TS SE and EMO with teachers’ perception of risk of contracting SARS-CoV-2 (CRP) and perception of health measures effectiveness (PEHM).

Furthermore, a higher level of JS implies a lower perception of health measures effectiveness.

Again, as in the previous model, we find an indirect path between TS, EMO, SE and teachers’ perceptions of COVID-19 pandemic impact on relationships through teachers’ risk perception of contracting SARS-CoV-2 (CRP) and perception of health measures effectiveness (PEHM).

In this case, it is evident that risk perception and health measures had a strong impact on the relationships that teachers had with parents, students, and colleagues.

Pandemic-specific stressors and ongoing emotional overload encouraged the search for coping strategies to look on the bright side and/or to find new opportunities, social and emotional support, physical exercise, and hobby activities [92].

Several studies reveal how important coping strategies are for school administrators and teacher trainers, helping to increase educators’ understanding of work-related stress and its connection with work manageability [89,90].

Teacher emotion, job satisfaction, and self-efficacy seem to be associated with support absence by school administrators and with a substantial increase in relationship workload due to the need for new teaching methods [89,90,92].

### 4.3. Differences across School Levels

Consistent with other research, our work confirms the importance of psychological support within schools. The multi-group path analysis showed that in both models there were no significant differences in the structural parameters across school levels.

This leads us to think that difficulties perceived by teachers working at different levels were similar, or at least not different, even when facing an emergency.

The results of this multi-group path analysis highlight the presence of help requests in schools; actually, Italy is one of the few countries that does not have a school psychologist.

During the pandemic, the profession of psychologist was integrated for the first time in schools by “MI prot. 23072” (N. d. T.: a Note by “MIUR”: Italian Ministry of Education, Universities and Research”) issued on 30 September 2020, and only for the period going from September to December 2020. That professional figure is now provided for in combined provisions (cd.) of Art. 1, paragraph 697, of Law Nr. 234/2021. Therefore, the issue of this law highlights a non-recognition of the professional figure of a school psychologist. It would therefore be necessary to verify and monitor possible effects of school psychologist’s presence on student’s well-being during this recovery phase.

In order to offer teachers more support it is necessary for school administrators, students and parents to search for social relationships and teaching methods.

### 4.4. Limitations and Future Work

The first limitation of this study is that participants’ replies were self-reported, an aspect that is considered a limitation when we consider the emergency.

In fact, teachers completed the survey once back at school in a delicate period that could have negatively affected the considerations and, consequently the responses to our survey. Furthermore, the sample is only made up of 12.4% males, this could also be due to the lack of male teachers in Italian schools.

Secondly, the absence of a base theory to refer to predict possible relationships and patterns, led us to choose and bring together the fundamental variables indicated by the literature. For this reason, this research should be read as data driven and useful as a basis for building new theories. The ultimate goal of our work and future work must be to build precisely the conceptual foundations that are useful for understanding the health status of schools during a period of emergency. This work can facilitate timely action by the authorities during emergency situations in order to structure coping interventions that are useful to teachers and indirectly also to students and their learning.

The last limitation, since path analyses do not prove causality but only help to select or infer from causal hypotheses, results of this study should not be considered as exhaustive and definitive but rather as a suggestion for future studies aimed at identifying causal relationships among variables [93].

## 5. Conclusions

During the COVID-19 period, teachers have experienced great difficulties in terms of teaching methods (ITM) and school relations (ISR). For this reason, it is essential to find innovative resources and strategies that are useful to Italian schools. The present study suggests a path could be particularly helpful regardless of the school level where teachers practice their profession.

In this research we verify that stress and cognitive factors (self-efficacy, emotions, job satisfaction) have positive correlations with teaching methods (ITM) and social relationships at school (ISR) (Hypotheses 1). Furthermore, we ascertained the existence of a causal link between social relationships at school (ISR) and teaching methods (ITM) on the constructs of teachers’ stress (TS), job satisfaction (JS), self-efficacy (SE), and emotional intensity at work (EMO). During the pandemic, that link was mediated by variables such as: teachers’ perception of risk of contracting SARS-CoV-2 (CRP) and perception of the effectiveness of health measures (PEHM) (Hypotheses 2). In contrast to what we expected, we did not find any difference in sensitivity between the primary school group and the secondary school group and much less among teachers of different ages (Hypothesis 3).

The present research motivates us to reflect on the preventive measures that could be taken to deal with the findings reported here. We need to strengthen teacher well-being if we intend to avoid the devastating effects of teacher burnout [94].

Considering the stress and risk factors perceived by Italian teachers, daily actions supporting teachers, enhancing positive emotions and self-efficacy are deemed necessary.

Institutions, public social and educational policies should take decisive actions aimed at recognizing the figure of a school psychologist and integrating it within a school.

For this reason, research in this area should continue to verify the effects of having permanent and actual psychological support when facing critical events such as pandemics.

## Figures and Tables

**Table 1:** Respondent profile.

Characteristics	Category	Frequency	Percentage (%)
Gender	Male	304	12.4
	Female	2142	87.6
Age	<40 years	577	23.6
	41–50 years	770	31.5
	51 years or above	1099	44.9
Education level	High school degree	576	23.5
	Bachelor’s degree	145	5.9
	Master’s degree	1372	56.1
	Specialization	353	14.4
Experience	Under 5 years	356	14.6
	between 5 and 10	386	15.7
	between 11 and 20	747	30.6
	over 21 years	956	39.1
Teaching Level	Preschool	317	13
	Primary school	843	34.5
	Lower secondary	585	23.9
	Higher secondary	701	28.7
Profession type	Curricular teachers	2100	85.9
	Special needs teachers	346	14.1
Teaching Location	Northern Italy	1570	64.2
	Centre Italy	419	17.1
	Southern Italy	457	18.7
Implementation of DE	Yes	1459	59.6
	No	987	40.4

Note: Age of children in: Preschool 3–5 y; Primary school 6–10 y; Lower secondary 11–13 y; Higher secondary 14–19 y.

**Table 2:** Bivariate correlations between variables.

Variable	Range	Mean (sd)	2.	3.	4.	5.	6.	7.	8.
1. Impact on teaching	14–140	97.5 (31)	0.60 **	0.17 **	−0.11 **	−0.12 **	0.14 **	0.20 **	−0.01
2. Impact on relations	4–40	26.6 (9)		0.16 **	−0.10 **	−0.08 **	0.13 **	0.24 **	−0.03
3. Stress	10–50	19.4 (7.9)			−0.33 **	−0.18 **	0.27 **	0.12 **	−0.09 **
4. Job satisfaction	5–35	21.7 (5.9)				0.29 **	0.02	−0.02	0.17 **
5. Self-efficacy	54–216	168.4 (27)					0.02	0.12 **	0.13 **
6. Emotional intensity	39–150	80.1 (10.1)						0.14 **	0.01
7. Risk perception	12–120	76.6 (18.8)							−0.01
8. Effectiveness of H.M.	7–70	48.9 (10.9)							1

Note: ** *p* < 0.01.

**Table 3:** Fit indexes of Model 1.

Group	*N*	*χ* ^2^	df	*p*-Value	RMSEA	SRMR	CFI	NNFI
Total	2015	1.958	3	0.058	0.000	0.007	1.000	1.007
Primary school	793	6.578	3	0.087	0.039	0.017	0.993	0.948
Lower secondary	555	0.939	3	0.816	0.000	0.007	1.000	1.052
Higher secondary	667	7.551	3	0.056	0.048	0.021	0.984	0.885
<40 years	482	0.495	3	0.920	0.000	0.007	1.000	1.086
40–50 years	623	0.817	3	0.845	0.000	0.007	1.000	1.048
>50 years	910	4.764	3	0.190	0.025	0.015	0.996	0.974

Note: RMSEA: root mean square error of approximation; SRMR: standardized root mean square residual; NNFI: non-normed fit index; CFI: comparative fit index.

**Table 4:** Multi-group path analysis of Model 1.

Model Invariance	*χ* ^2^	df	*p*-Value	Delta *χ*^2^	Delta df	*p*-Value	RMSEA	SRMR	NNFI	CFI	ΔCFI	BIC	ΔBIC
**School levels**													
Baseline model	15.069	9	0.089				0.032	0.014	0.959	0.994		113,075	
Metric invariance	64.148	37	0.004	49.079	28	0.008	0.033	0.032	0.955	0.974	−0020	112,911	−164
Partial metric Invariance	39.976	31	0.130	24.907	22	0.302	0.021	0.024	0.982	0.991	−0.003	112,933	−142
**Teacher age**													
Baseline model	6.076	9	0.732				0.000	0.010	1.020	1.000		113,040	
Metric invariance	40.448	37	0.321	34.372	28	0.189	0.012	0.026	0.994	0.997	−0.003	112,861	−179

Note: RMSEA: root mean square error of approximation; SRMR: standardized root mean square residual; NNFI: non-normal fit index; CFI: comparative fit index.

**Table 5:** Fit indexes of Model 2.

Group	*N*	*χ* ^2^	df	*p*-Value	RMSEA	SRMR	CFI	NNFI
Total	2351	1.486	3	0.685	0.000	0.006	1.000	1.009
Preschool	302	0.860	3	0.835	0.000	0.011	1.000	1.099
Primary school	809	6.468	3	0.091	0.038	0.016	0.993	0.950
Lower secondary	566	1.162	3	0.076	0.000	0.008	1.000	1.048
Higher secondary	674	7.281	3	0.063	0.046	0.020	0.984	0.891
<40 years	564	1.309	3	0.727	0.000	0.009	1.000	1.047
40–50 years	739	1.830	3	0.608	0.000	0.009	1.000	1.024
>50 years	1048	3.756	3	0.289	0.016	0.013	0.999	0.991

Note: RMSEA: root mean square error of approximation; SRMR: standardized root mean square residual; NNFI: non-normed fit index; CFI: comparative fit index.

**Table 6:** Multi-group path analysis of Model 2.

Model Invariance	*χ* ^2^	df	*p*-Value	Delta χ^2^	Delta df	*p*-Value	RMSEA	SRMR	NNFI	CFI	ΔCFI	BIC	ΔBIC
*School levels*													
Baseline model	15.770	12	0.202				0.023	0.013	0.978	0.997		126,125	
Metric invariance	86.026	54	0.004	70.256	42	0.004	0.032	0.034	0.958	0.973	−0.024	125,869	−256
Partial metric Invariance	42.208	36	0.220	26.438	24	0.331	0.017	0.024	0.988	0.995	−0.002	125,965	−160
*Teacher age*													
Baseline model	6.895	9	0.648				0.000	0.010	1.013	1.000		125,925	
Metric invariance	52.222	37	0.050	45.327	28	0.020	0.023	0.026	0.977	0.987	−0.013	125,753	−172
Partial metric Invariance	35.153	31	0.278	28.258	22	0.167	0.013	0.019	0.993	0.996	−0.004	125,783	−143

Note: RMSEA: root mean square error of approximation; SRMR: standardized root mean square residual; NNFI: non-normed fit index; CFI: comparative fit index.

## Data Availability

The datasets that were generated for this study are available upon request from the corresponding author.
